# Sub-Axial Cervical Facet Dislocation: A Review of Current Concepts

**DOI:** 10.7759/cureus.12581

**Published:** 2021-01-08

**Authors:** Islam Mubark, Amr Abouelela, Mohammed Hassan, Ahmed Genena, Neil Ashwood

**Affiliations:** 1 Trauma and Orthopaedics, University Hospitals of Derby and Burton NHS Foundation Trust, Derby, GBR; 2 Trauma and Orthopaedics, Faculty of Medicine, Helwan University, Helwan, EGY; 3 Trauma and Orthopaedics, James Paget University Hospitals NHS Foundation Trust, Norwich, GBR

**Keywords:** subaxial, facet dislocation, cervical fusion, cervical trauma, spine injury, emergency management, decision making, surgical management

## Abstract

Cervical facet dislocation is a serious injury that carries risks of short- and long-term morbidity. The optimal management of these injuries remains controversial with the ongoing debate regarding indications and requirements for closed reduction, timing, type of surgical approach and method of fixation. This review gives an update on the relevant anatomy, classification systems for sub-axial cervical facet dislocation and an overview of the current concepts regarding their management, including surgical approaches and the choice of implants.

## Introduction and background

Traumatic sub-axial cervical spine facet joint dislocation is characterized by unilateral or bilateral facet dislocation between C3 and C7 cervical vertebrae causing displacement of one cervical vertebra relative to another [1]. It is usually the result of combined flexion and distraction moments most commonly caused by a road traffic accident [2]. There has been diversity in the literature in describing the best approach for diagnosis and management.

## Review

Anatomy and biomechanics of injury

**Figure 1:**
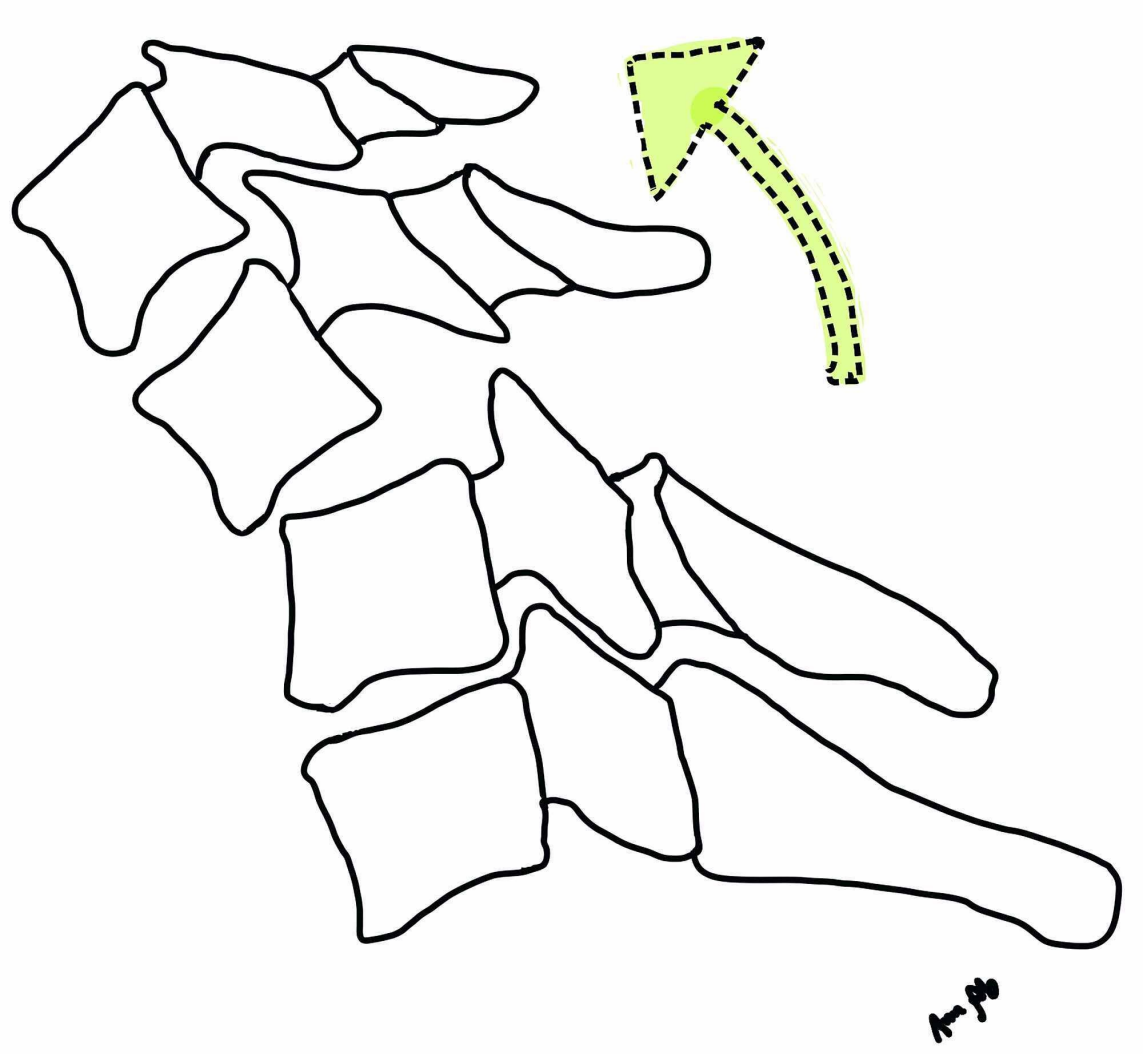
Illustration demonstrating flexion distraction injury as the usual mechanism for facet dislocation

The cervical spine can be divided into two main regions: the craniocervical junction, from the occiput (C0) joint to the axis (C2), and the sub-axial cervical spine, which includes injuries from C3 to C7 [3]. Almost two-thirds of cervical spine injuries occur within the sub-axial cervical spine, with dislocations occurring most commonly at C5-C6 and C6-C7 levels [4]. Cervical facet dislocation usually occurs as a result of combined flexion and distraction forces (Figure 1).

An element of rotation may lead to unilateral rather than bilateral facet dislocation [5]. These forces lead to variable disruption of the ligamentous structures such as the longitudinal ligaments, ligamentum flavum, apophyseal joint ligaments, the annulus fibrosis and the interspinous ligaments. The bony injuries could involve facet fractures, lamina fractures and endplate fractures [6]. There is a significantly higher disruption of the osteo-ligamentous structures with bilateral facet dislocations as compared to unilateral facet dislocations and predictably associated with a higher incidence of neurological injury [7].

Classifications

In 1982, Allen et al. published their classical paper describing the mechanistic classification of cervical spine fractures and dislocations [5]. They classified facet dislocations under the flexion-distraction injury. This injury is divided into four stages starting with facet subluxation to complete dislocation (100% displacement). In 2007, Vaccaro et al. published the new Sub-axial Cervical Spine Injury Classification System (SLIC) (Table 1). It aims to incorporate injury pattern, severity and neurological insult to define injury prognosis and guide treatment [8].

**Table 1:** The Sub-axial Cervical Spine Injury Classification System (SLIC)

Characteristics	Points
Injury morphology	
No abnormality	0
Compression	1
Burst	2
Distraction	3
Translation	4
The Integrity of disco-ligamentous complex	
Intact	0
Indeterminate	1
Disrupted	2
Neurological status	
Intact	0
Nerve root injury	1
Complete	2
Incomplete	3
Persistent cord compression	+1

By default, all cases with cervical facet dislocation/subluxation will achieve enough score to warrant operative treatment. In 2016, the AO (Arbeitsgemeinschaft für Osteosynthesefragen) organization in collaboration with Vaccaro et al. reached a consensus over the classification of sub-axial cervical spine injuries [9]. They provided a newer classification system of cervical spine fractures merging the SLIC with the traditional ABC classification scheme with group C representing injuries with displacement or translation of one vertebral body relative to another. It added the subgroup F to represent facet injury. The classification also takes into consideration neurological injury and presence of what the AO names case-specific modifiers such as posterior ligamentous complex (PLC) injury and disc herniation.

Diagnosis and clinical evaluation

Cervical facet dislocation is usually the result of a high-trauma injury mostly sustained from road traffic accidents. It is not uncommon to be associated with other multiple injuries. Initial management and resuscitation should be in line with the Advanced Trauma Life Support (ATLS) protocol, and emergent injuries should be treated first in the order of priority [2]. Clinicians in emergency service can use assessment tools such as the Canadian C-spine rules or the National Emergency X-Radiography Utilization Study (NEXUS) rules to define patients with risk of blunt cervical trauma [10,11]. Both tools use a combination of history and clinical examination findings to stratify patient risk from no risk to high risk. Both of these clinical tools have proved sensitivity ranging from 90% to 100% in several studies [12]. Documentation of patient neurology should follow a standardized hospital format to facilitate handover between different clinicians. The most commonly utilized tool is the International Standards for Neurological Classification of Spinal Cord Injury (ISNCSCI), commonly referred to as the ASIA Exam, developed by the American Spinal Injury Association (ASIA) [13].

Imaging

**Figure 2:**
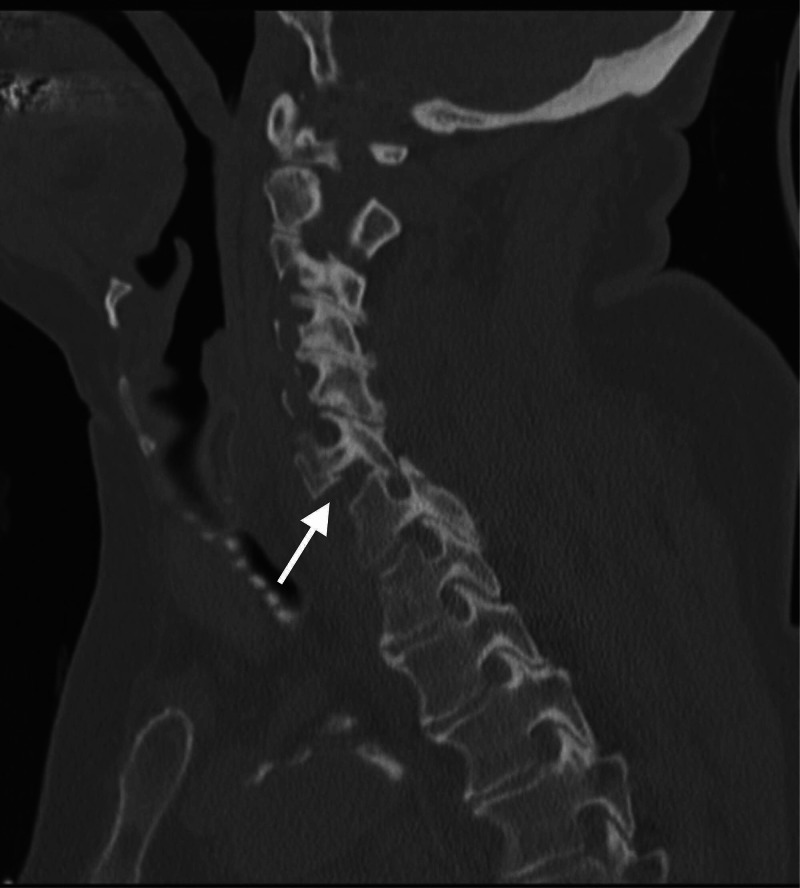
Sagittal cuts of the cervical spine CT scan demonstrating unilateral facet dislocation at the C6-C7 level Case courtesy of Associate Prof. Frank Gaillard, https://radiopaedia.org/, rID: 35609

Radiological clearance of a cervical spine injury varies between hospitals depending on their local protocols and availability of resources. An adequate plain radiograph may show the displacement of vertebral bodies relative to each other. Bilateral facet dislocation usually leads to higher degrees of displacement compared to unilateral dislocation. It is sometimes difficult to appreciate the facet joint injury in cases without frank dislocation but the percentage of overlap of the facet joint surfaces of two neighbouring articular processes can indicate an unstable lesion of the facet joint. If the articulating surfaces overlap less than 50% of their length, the joint is considered to be unstable. A CT scan of the whole cervical spine is becoming more commonly used in the initial assessment of suspected cervical trauma (Figure 2) [14].

**Figure 3:**
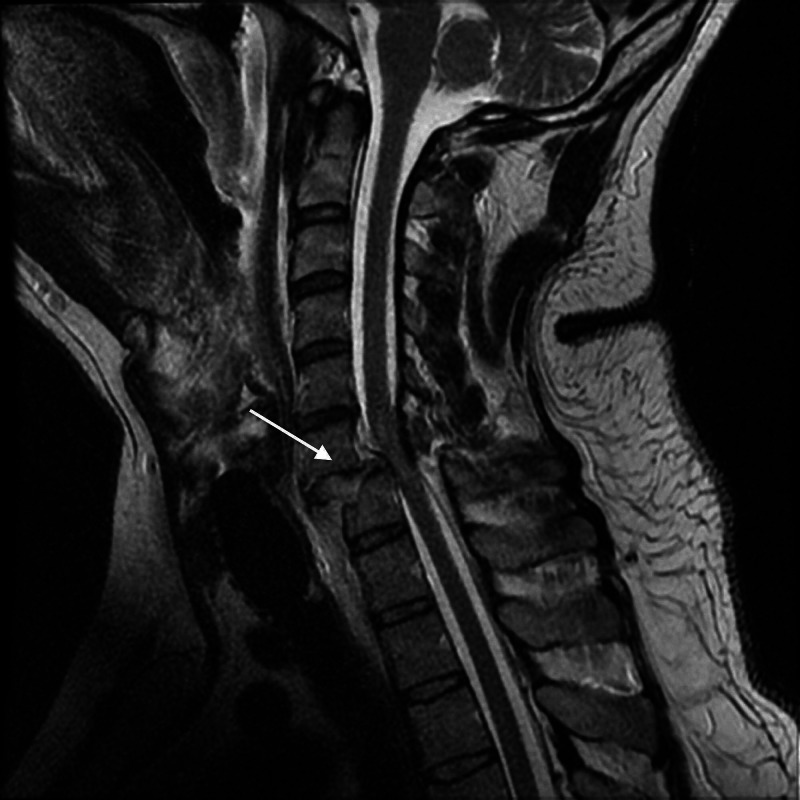
T2 sagittal cuts of an MRI (the same case as in Figure 2) demonstrating a complete rupture of the ligamentum flavum at C6-7 with related short-segment posterior epidural hematoma, and disrupted ALL at C7 with pre-vertebral hematoma, also showing the extent of damage to the intervertebral disc ALL, anterior longitudinal ligament Case courtesy of Dr Andrew Dixon, https://radiopaedia.org/, rID: 31837

It is available in the majority of trauma centres around the clock and has a high diagnostic value for bony as well as for most of the disco-ligamentous injuries [15,16]. The British Orthopaedic Association Standards for Trauma and Orthopaedics (BOASTs) in England recommended using a thin-slice (2-3 mm) helical CT scan from the base of the skull to at least T1 with both sagittal and coronal reconstructions. The MRI remains the most sensitive diagnostic tool to evaluate the soft tissues including associated disc herniation, ligament injury and traumatic cord injury (Figure 3) [17].

Problems with the routine use of MRI in the diagnosis of cervical facet dislocations relate to delayed timing of dislocation reduction and cost-effectiveness [18]. The use of MRI is mandatory for patients undergoing surgical open reduction and/or fixation as to plan for the approach of reduction and decompression of bony fragment or extruded disc material. If closed reduction is planned first, it is generally recommended to obtain an MRI first in uncooperative patients with altered mental status to define the status of the spinal cord and any disc or bony injury that may place the spinal cord at risk. Whether or not to perform MRI before a closed reduction in an awake examinable patient is still debatable [18].

Treatment

The treatment of facet dislocation aims to maintain the functional and anatomical integrity of the spinal cord throughout: restoring spinal canal alignment and achieving spinal stability [19]. These all should be done to stabilize or recover any neurological deficit caused by the injury and avoid long-term complications of pain, stiffness, and instability. To achieve these goals, reduction of the dislocated facets is required to allow for the healing of injured tissue, decompress neural elements and restore normal anatomy. Like any other joint dislocation, the reduction of facet dislocation can be achieved closed or open. Contraindications to closed reduction include an uncooperative or unexaminable patient where the neurological examination is not reliable as well as cases with imaging studies showing evidence of offending disc prolapse or bony fragments that can potentially compress the cord during reduction. However, the debate is still active regarding whether or not an MRI scan is always needed before the reduction in awake cooperative patients [20-22]. Several reports, including those by Vaccaro et al., have indicated that closed reduction in an awake and alert patient may be safe without obtaining a prereduction MRI. Some recent studies even have indicated that closed reduction in sedated patients may be safe in most cases [18,21,23].

Timing for closed reduction

As in any joint dislocation, the reduction should be done as soon as it is safe. This means a physiologically stable patient, a competent surgeon, and available equipment. There is no consensus on the timing for closed reduction. Newton et al. demonstrated that closed reduction of cervical dislocation within four hours of injury is important in achieving the desired outcome with delays longer than this being associated with a poor outcome. They reported that five out of eight patients, who had a successful reduction within four hours of injury, made a full recovery from complete paralysis [24]. Ahmed et al. reported a reduced rate of only 44% with around 6% risk of developing a transient neurological deficit. Authors attributed the less favourable results to the fact that 83% of reduction attempts were done 10 hours or later post-injury [25].

The technique of closed reduction

Closed reduction should be performed in the operating room, in intensive or high dependency units where vitals monitoring, resuscitation, medications, and equipment are readily available including traction kits, traction weights, and fluoroscopy machine. Patients should be transferred with full spinal precautions and logroll transfer. Patients are assessed neurologically before the start of traction to define baseline neurology. Analgesia and sedation can be used including opioids, benzodiazepines as well as antiemetics. Currently, the most commonly used device for skull traction is the tongs design introduced by Gardner in 1973. Using this spring-loaded tension device led to a simple and reliable application of the tongs [20,23]. The pins were then positioned just below the superior temporal line, avoiding the temporal muscle and the temporal artery. The newly developed Singhal Traction Bed uses a load cell tensioner handle to produce incremental traction with simultaneous flexion of the cervical spine [24].

The initial, subsequent, and maximum traction weight is variable between studies with traction weight up to 140 pounds being reported [20]. The use of an initial 2.5 to 5 kg traction weight followed by 2-5 kg for each level above dislocation seems to be accepted by many studies [25,26]. After starting initial traction weight, an X-ray should be taken to assess the dislocation level, and repeat neurological assessment is done. Incremental weights of 2-5 kg are added every 5-10 min depending on the relative position of the dislocated facets followed by X-ray screening and clinical assessment of neurological levels. When the facets start to unhinge, incremental traction can be combined with flexion to aid reduction. Once it is achieved, traction weights should be reduced to 10-20 kg and the neck should be slightly extended. In cases of unilateral facet dislocation, rotating the head 40° towards the side of dislocation can help reduce the facet dislocation. The outcome of traction can follow the three following scenarios: (1) the patient starts to develop a new neurological deficit; here traction weight should be reduced until the new neurological deficit resolves and then urgent MRI and transfer for open reduction should be planned; (2) maximum traction weight reached without reduction; here, traction should be reduced to 1-2 kg per level of vertebrae above the injury level and then urgent MRI and transfer for surgical open reduction should be planned; (3) successful reduction; traction in line with weights should be maintained using 7.5-10 kg and rigid collar; repeat full neurological examination and MRI can be done less urgently to plan for surgical fixation.

Surgical treatment

Open reduction and cervical fusion are indicated in cases of failed closed reduction and cases contraindicated for closed reduction. Even in cases with a successful closed reduction, it has become the standard care for treatment. The non-operative management of reduced facet dislocation in the form of halo traction and collar immobilization has fallen out of favour due to the reportedly high rates of instability, later disability in the terms of pain, delayed neurological injury and stiffness [27]. The options of surgical approach for unilateral or bilateral facet injuries include a stand-alone anterior approach, a stand-alone posterior approach, a combined anterior and posterior approach, or a staged anterior/posterior/anterior approach [28,29]. Which approach is used seems to be dependent on several factors: (1) degree of instability of injury, i.e. unilateral versus bilateral and degree of osteo-ligamentous injury; (2) the presence of anterior disc herniation; and (3) whether the dislocation is reducible from a single anterior or posterior approach alone.

Timing for surgery

The timing of the surgery is another controversial aspect of the surgical treatment of cervical facet dislocation. Factors such as failed closed reduction and/or progressive neurological deficits indicate for urgent surgery. In 1999, Mirza et al. recommended that surgical intervention following an acute cervical spinal cord injury should be within 72 hours following the injury to increase the chance of recovery of early neurological function and avoid delayed neurological recovery [30]. In 2016, the Surgical Timing in Acute Spinal Cord Injury Study (STASCIS) showed that early reduction and decompression (<24 hours) achieved a higher rate of neurological improvement compared with late decompression (>24 hours) [31].

Anterior cervical reduction and fusion

Anterior cervical decompression and fusion (ACDF) is indicated for cases where ruptured or extruded cervical disc jeopardizes the spinal cord and when anterior decompression is needed to reduce the risk of cord injury before facet reduction. It provides a relatively simple approach, less soft tissue damage and safer supine position in cases of polytrauma patients. It also allows for the restoration of cervical lordosis that favours the mechanical stability of the injured spine by reducing tension on commonly damaged PLC.

**Figure 4:**
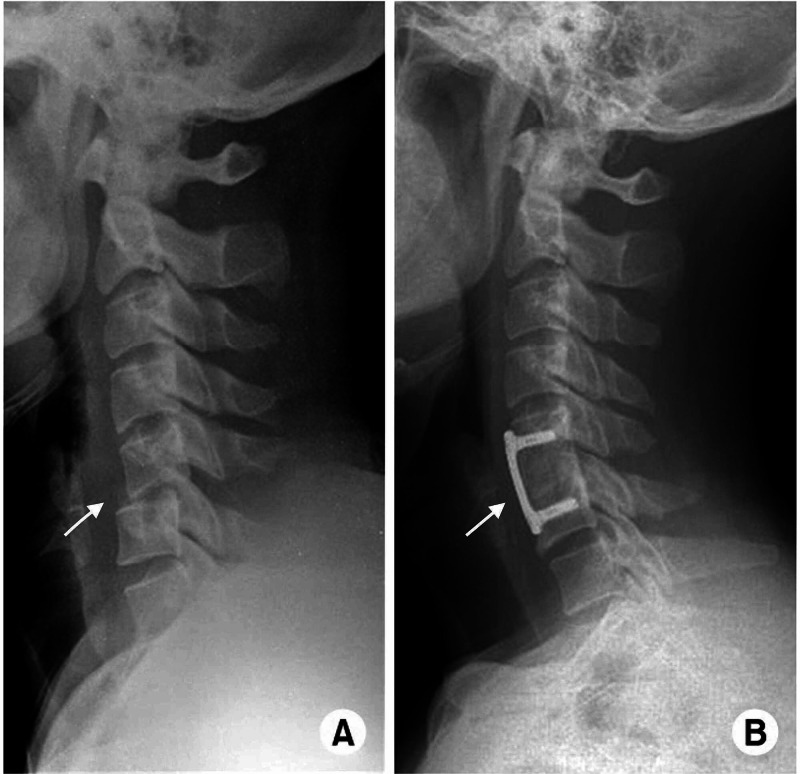
C5-6 unilateral facet joint dislocation. Lateral radiographs of the cervical spine demonstrating (A) unilateral facet joint dislocation at the C5-C6 level and (B) two years after operation (ACDF) showing well reduction and fixation state ACDF, anterior cervical decompression and fusion Image courtesy: ResearchGate. Available from https://www.researchgate.net/figure/C5-6-unilateral-facet-joint-dislocation-Lateral-radiographs-A-of-the-cervical-spine_fig14_274058208 (accessed November 25, 2019), via license: Creative Commons Attribution-NonCommercial 3.0 Unported.

The patients are positioned supine and the standard cervical approach is used for the defined level of dislocation. It is better to completely visualize the extent of disc extrusion for adequate decompression before the trial of reduction. As the rostral dislocated vertebra's lower endplate may block the view to the disc space, removal of a portion of the ventrocaudal aspect of the rostral vertebral body may be needed for full visualization [32]. After adequate discectomy, a reduction can be safely achieved via different techniques. Most commonly, vertebral body posts such as Caspar pins can be placed in both rostral and caudal vertebral body and linked to distraction device. They can be placed divergent to add bending moment with distraction. Using manual or blunt compression to push the rostral vertebra backwards can aid in reduction. In cases of unilateral facet dislocation, the pins can be applied at an angle to each other in the coronal plane to allow for rotation during reduction [33]. Another technique describes using a blunt instrument inserted in the superior endplate of the caudal vertebra and used as leverage to push the rostral vertebra backwards [34]. An alternative manoeuvre for reducing a locked facet joint is continuous intraoperative external cranial traction. After achieving reduction and confirming with X-rays, the next step is to fix the injured cervical level. Different techniques have been described, including conventional plates, locking plate system, bone graft, and disc cages, for the fusion of the disk space. Using locking plates plus disc space fusion with cages or bone graft has become the most popular technique for ACDF in facet dislocation in the last two decades [35-37] (Figure 4).

It provides a stable rigid fixed-angle construct against the deforming kyphotic forces on the cervical spine [38,39]. There have been concerns regarding using isolated anterior ACDF only for bilateral facet dislocation as the amount of posterior ligamentous injury and nearly complete loss of the tension band effect of the PLC may result in loss of reduction and postoperative kyphosis [40]. Johnson et al. reported a loss of postoperative cervical alignment in 13% of 87 patients with fracture facet subluxation treated by ACDF due to mechanical failure of posterior elements, especially in distractive lesions [41]. These outcomes seem to be related to the extent of bony injury associated with the facet dislocation where additional facet fracture and/or endplate fractures increase the risk of mechanical failure of standalone anterior fusion. Anissipour et al. reported that of 36 patients with facet dislocations treated with ACDF using a fixed locking plate, 16 were unilateral and 20 were bilateral. There were only three treatment failures (8%); all three had an associated endplate fracture and one of them had an additional facet fracture. The authors concluded that in the absence of an endplate fracture, ACDF is a reasonable treatment option in patients with single-level cervical facet dislocation. They advised using longer screws within 2 mm of the posterior vertebral cortex in a locking fashion and accentuating cervical lordosis as measures to decrease the risk of mechanical failure [42].

Posterior cervical reduction and fixation

Posterior cervical reduction and fixation have the advantages of direct visualization and reduction of dislocation, and decompression of any offending bony fragment compressing the cord such as a lamina fragment into the canal [43,44]. It allows for biomechanically stronger fixation of the tension side of the spine especially in osteoporotic bones [44]. Unilateral facet dislocation is more difficult to reduce and sometimes they are locked and more likely to need direct posterior reduction. The patient is positioned prone and a standard posterior approach is utilized. Reduction of a dislocated facet can be achieved by using external traction - leverage of the inferior facet of a dislocated vertebra over the superior facet of the caudal one using a blunt instrument. In cases of irreducible locked facet, removing the zygapophyseal apex of the lower vertebra can assist in reduction [45]. Posterior fixation can be achieved using wire ropes, lateral mass screws, or pedicular screws. The posterior reduction can risk neurological deterioration in cases of anterior compressive lesions such as disc herniation. This has always been considered as an absolute contraindication. However, reports about the frequency of this deterioration after posterior open reduction are scarce. Nakashima et al. reported a series of 40 patients with traumatic cervical herniation treated by a posterior approach without the need for anterior cervical surgery with no neurological deterioration observed after posterior open reduction, suggesting that the risk of neurological deterioration may be less than previously thought [43]. However, there is still no strong current evidence to exclude the possibility of postoperative neurological deterioration, and thus, preparations for anterior supplemental surgery should always be made in these categories of patients. Another concern with the posterior approach is an increased risk of wound complications compared with anterior surgery. It may sometimes fail in restoring cervical lordosis especially in cases with endplate fractures and disc space damage with long-term risk of pain, instability, and loss of fixation. Polytrauma and clinically unstable patients may also have problems with an operation in the prone position [46].

Combined anterior and posterior cervical approach

The surgeon may have to stage fixation of the spine in a different way (anterior-posterior, posterior-anterior, anterior-posterior-anterior). In cases of failed reduction through an anterior approach, the surgeon may have to turn the patient to prone position for posterior reduction followed by anterior ACDF. Furthermore, after successful reduction and fixation through either posterior or anterior approach, the surgeon can add combined anterior or posterior fixation to achieve robust fixation with higher rates of union. It is also indicated where the mechanical stability of the construct is at question especially in osteoporotic bones, ankylosing spondylitis, and in the case of associated endplate and facet fractures [47]. The drawback with combined approaches is the increased iatrogenic surgical risk of injury and further restricted spinal movement.

## Conclusions

The optimum treatment strategy of cervical facet dislocation is still a matter of debate. Despite agreement in the literature over the role of closed reduction and surgical treatment of these injuries, there are still areas of debate including indications for MRI, MRI timing, and superiority of one surgical approach over another.
